# Cross‐cultural differences in Parkinson's disease caregiving and burden between the United States and Mexico

**DOI:** 10.1002/brb3.1753

**Published:** 2020-07-18

**Authors:** Erin R. Smith, Paul B. Perrin, Carmen M. Tyler, Sarah K. Lageman, Teresita Villaseñor

**Affiliations:** ^1^ Department of Psychology Virginia Commonwealth University Richmond VA USA; ^2^ Department of Physical Medicine and Rehabilitation Virginia Commonwealth University Richmond VA USA; ^3^ Department of Neurology Parkinson's & Movement Disorders Center Virginia Commonwealth University Richmond VA USA; ^4^ Master of Neuropsychology Neurosciences Department University of Guadalajara Guadalajara Mexico

**Keywords:** burden, caregiving, cross‐cultural, Mexico, Parkinson's disease, United States

## Abstract

**Introduction:**

Given the rapidly aging population in both the United States and Mexico, rates of Parkinson's disease (PD) are likely to rise in both countries, suggesting that the number of individuals providing informal care will also increase, and the healthcare system will have to consider the burden this places upon caregivers. Therefore, the purpose of the current study was to examine differences in PD caregiving and burden between the United States and Mexico.

**Methods:**

Data were collected from PD caregivers in the Parkinson's Clinic at the Hospital Civil Fray Antonio Alcalde in Guadalajara, Mexico (*N* = 148) and the Parkinson's and Movement Disorders Center at Virginia Commonwealth University in Richmond, Virginia (*N* = 105) regarding caregiver demographics and self‐reported burden.

**Results:**

Despite considerably more time spent in caregiving duties, higher rates in unemployment or underemployment, and lower education levels, Mexican PD caregivers reported significantly less personal strain and role strain than did their United States counterparts. Even after controlling for these and other demographic differences between the two sites, the differences in caregiver burden remained.

**Conclusions:**

Latino cultural values in Mexico encouraging the importance of caring for family members with PD and respecting elders may promote caregiving and even make it a point of cultural pride, helping to overcome potential negative effects on caregivers seen in the United States. The scientific and medical communities should view caregiving as a culturally embedded and potentially positive role, rather than predominantly as burdensome as frequently conceptualized in Western or Eurocentric cultures.

## Graphical abstract

The purpose of this study was to examine differences in Parkinson's disease (PD) caregiving and burden between the United States and Mexico. Despite considerably more time spent in caregiving duties, higher rates in unemployment or underemployment, and lower education levels, Mexican PD caregivers reported significantly less personal strain and role strain than did their United States counterparts. Even after controlling for these and other demographic differences between the two sites, the differences in caregiver burden remained.

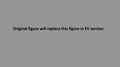

## 1 INTRODUCTION

Parkinson's disease (PD) is a neurodegenerative illness affecting 1–2 individuals per 1,000 of the population (Tysnes & Storstein, 2017), or about 1% of those in the 65–69 age range and 1% to 3% of those aged 80 and above (Nussbaum & Ellis, 2003). Prevalence rates in Mexico for those aged 65 and older are similar at approximately 3.14 individuals with PD per 1,000 (Rodriguez‐Violante, Velásquez‐Pérez, & Cervantes‐Arriaga, 2019). As increased age is the principal risk factor for developing PD and the exacerbation of PD symptoms (Hindle, 2010), the rising growth of the aging population in Latin America (Wong & Palloni, 2009) highlights the importance of research on PD in this region. In fact, rates of PD have already shown increases in Latin America (GBD 2016 Parkinson's Disease Collaborators, 2018), with some indications that PD prevalence in Mexico will double within 20 years (Cantu‐Martinez et al., 2014). However, to date, only a few studies have focused on PD in Latin America (e.g., Rodríguez‐Violante, Camacho‐Ordoñez, Cervantes‐Arriaga, González‐Latapí, & Velázquez‐Osuna, 2015), and despite the trend of increasing PD diagnoses, research on Latin American PD patients and their caregivers continues to be sparse (Trapp, MacKenzie, Gonzalez‐Arredondo, Rodriguez‐Agudelo, & Arango‐Lasprilla, 2019).

Parkinson's disease is associated with gradual progression of physical symptoms (Shulman et al., 2016) and cognitive dysfunction (Petrou et al., 2015). Although PD is classified primarily as a movement disorder with motor symptoms typified by bradykinesia, resting tremor, and rigidity (Tysnes & Storstein, 2017), the neurodegeneration of specific regions of the brain involved in PD also leads to substantial neurobehavioral symptoms (Mandir & Vaughan, 2000). These nonmotor symptoms of PD may include depression, cognitive impairment (e.g., dementia, executive functioning), apathy, sleep, and anxiety (Mosley, Moodie, & Dissanayaka, 2017).

As symptoms become more severe, the individual's ability to function independently lessens, often resulting in a loss of autonomy and necessitating increasing reliance on assistance from a caregiver (Shulman et al., 2008). Provision of this care may eventually entail loss of outside employment, sacrifice of social engagement, and a reduction in self‐care for the care provider (Bhimani, 2014). Over time, responsibilities of caregiving have been associated with a negative psychological state, termed caregiver *burden* (Mosley et al., 2017). A number of studies have identified associations between motor symptoms and caregiver burden (Lau & Au, 2011), but increasing attention has recently been given to the nonmotor symptoms of PD, which have also been associated with caregiver burden (Mosley et al., 2017). Research has suggested that caregiver burden is a particularly important construct among Mexican PD caregivers and has been linked directly with family dynamics and caregiver health‐related quality of life (Trapp et al., 2019).

To date, very little research has examined PD in regions outside of North America and Europe. The majority of research conducted outside of these regions has focused on data derived from medical records or drug consumption data (Pringsheim, Jette, Frolkis, & Steeves, 2014). This is problematic for low‐ to middle‐income countries, as these estimates inherently exclude individuals who are unable to obtain medical care or prescription drugs to treat PD (de Rijk et al., 1997). Further, these studies have also not considered the unique culturally determined treatment practices and varying access to care for PD throughout the world (Chiò, Magnani, & Schiffer, 1998). As such, there is a critical need to examine PD caregiving in diverse regions of the world, such as Latin America. Given the rapidly aging population in both the United States (Marras et al., 2018) and Mexico (Cantu‐Martinez et al., 2014), rates of PD are likely to rise in both countries, suggesting that the number of individuals providing informal care will also increase.

Inclusion of individuals living with PD in research from different cultural contexts may lead to early identification and appropriate interventions to address caregiver burden (Carod‐Artal, Mesquita, Ziomkowski, & Martinez‐Martin, 2013). Further, a more in‐depth understanding of the lived experiences of PD caregivers may serve to better support individuals with PD and their families through evidence‐based interventions. With rising rates of PD throughout the world (Pringsheim et al., 2014), cross‐cultural comparisons of PD caregiver characteristics and burden may lead to important insights for easing psychosocial burdens associated with caregiving. Very little research has made comparisons between individuals with PD in any part of Latin America and the rest of the world. A review of the literature yielded one study that examined a registry of individuals living with PD in Mexico. Analyses of this registry found that participants were of similar age to individuals in registries from other countries (Cervantes‐Arriaga et al., 2013). However, individuals in the registry were less educated, had a longer period from the onset of PD symptoms to diagnosis, and did not use dopamine agonists as frequently to address PD symptoms (Cervantes‐Arriaga et al., 2013). Therefore, the purpose of the current study was to examine differences in PD caregiving and burden between the United States and Mexico. We hypothesized that there would be significant mean differences in (a) caregiver characteristics and (b) caregiver burden between the United States and Mexico. Furthermore, with more access to specialized PD treatments in the United States, it was hypothesized that caregivers at the United States site would report lower levels of burden than caregivers at the Mexico site. Alternatively, there was a possibility that with predominant cultural values such as familismo and marianismo, caregivers at the Mexico site would report lower levels of caregiver burden.

## 2 METHOD

### 2.1 Participants

Data were collected from PD caregivers in the Parkinson's Clinic at the Hospital Civil Fray Antonio Alcalde in Guadalajara, Mexico (*N* = 148) and the Parkinson's and Movement Disorders Center at Virginia Commonwealth University in Richmond, Virginia (*N* = 105), two urban PD specialty clinics in academic medical centers situated in state capitals. Eligibility requirements for participation in this study included (a) identifying as a caregiver for an individual with a PD diagnosis, (b) being 18 years old or older, and (c) fluency in Spanish for the Mexico site or English for the United States site. Please see Table 1 for caregiver and care recipient demographic characteristics.

**TABLE 1:** Characteristics of PD caregivers and care recipients (*N* = 253)

Demographic variable	Value		
	United States	Mexico	*p*‐value
CG age, years, mean (*SD*)	68.73 (8.36)	53.66 (14.96)	<.001
CG sex (%)			
Female	68.6	76.4	.169
Male	31.4	23.6	
CG relationship status (%)			
Married or partnered	96.2	77.7	<.001
Single	1.0	17.6	
Widowed	0	0.7	
Divorced or separated	2.9	4.1	
CG education (%)			
No formal schooling	0	4.7	<.001
Elementary school	0	58.1	
High school/GED	25.7	5.4	
2‐year/technical degree	11.4	13.5	
4‐year college degree	33.3	16.2	
Master's degree	21.9	2.0	
Doctorate degree	7.6	0	
CG social class (%)^a^			
Lower class	0	15.5	<.001
Working class	9.5	24.3	
Lower middle class	23.8	37.2	
Upper middle class	63.8	22.3	
Upper class	2.9	0.7	
CG relationship to individual with PD (%)			
Parent	3.8	34.5	<.001
Aunt/Uncle	1.0	1.4	
Spouse/romantic partner	93.3	51.4	
Sibling	0	7.4	
Child	0	0	
Friend	1.9	0.7	
Professional caregiver	0	0	
Cousin	0	0.7	
Other	0	4.1	
Number of individuals who assist in providing care, mean (*SD*)	0.46 (1.08)	0.62 (0.88)	.186
Months providing care, mean (*SD*)	46.78 (81.33)	52.38 (49.22)	.501
Hours per week of care, mean (*SD*)	59.38 (64.56)	107.39 (61.34)	<.001
CG current occupation (%)			
Homemaker (Mexico only)	0	14.2	<.001
Full‐time employment	16.2	12.2	
Part‐time employment	8.6	28.4	
Student	0	0.7	
Unemployed	5.7	22.3	
Retired	64.8	6.1	
Other	4.8	16.2	
CR age, years, mean (*SD*)	71.61 (8.13)	65.68 (10.78)	<.001
CR sex (%)			
Female	35.2	48.0	.044
Male	64.8	52.0	
Months since PD diagnosis, mean (*SD*)	92.25 (82.84)	63.22 (60.88)	.002

Abbreviations: CG, caregiver; CR, care recipient; PD, Parkinson's disease.

^a^ Social class categories were selected as presented in this table in response to the question “What is your social class?”

### 2.2 Measures

#### 2.2.1 Demographic information

Participants provided data regarding their age, sex, romantic relationship status, relationship to individual with PD, number of individuals who assist in providing care, months providing care, hours per week of care, and current occupation. They also reported on the age, sex, and months since PD diagnosis of the care recipient.

#### 2.2.2 Caregiver Burden

Caregiver burden was assessed with the 12‐item Zarit Burden Inventory (ZBI) (Bedard et al., 2001). Participants select a response from 0 (Never) to 4 (Nearly Always) on a Likert‐type scale, with higher scores corresponding to higher caregiver burden. Scores from each item are combined to create a total score ranging from 0 to 48. The ZBI (full version) has been validated for PD caregivers (Martinez‐Martin et al., 2007) and also for Spanish speakers (Marin, 1996). The ZBI has two subscales which were used in the current study: Personal Strain (level of personal stress due to caregiving) and Role Strain (level of stress due to overload or role conflict). The scale for both samples in the current study showed good internal consistency (Mexico *α* = .86; United States *α* = .91).

### 2.3 Procedure

Institutional Review Boards at Virginia Commonwealth University and Hospital Civil Fray Antonio Alcalde reviewed and approved the current study's research protocol. Research assistants used written and verbal advertising to recruit participants while they were in waiting rooms or after clinician referral post a medical appointment. Informed consent was obtained from each participant prior to study enrollment. Eligible participants in the United States completed all study measures using pencil and paper, but due to the likely higher rate of illiteracy at the Mexico site, the study measures were administered orally by study staff.

### 2.4 Data analyses

Potential demographic and burden differences between sites were analyzed using analyses of variance (ANOVAs), chi‐square tests, and analyses of covariance (ANCOVAs) as appropriate.

## 3 RESULTS

### 3.1 Demographic differences

Caregivers at the United States site were significantly older than caregivers at the Mexico site, *F*(1, 251) = 87.133, *p* < .001. There was a similar gender distribution for caregivers between sites, *χ* ^2^ (1) = 1.892, *p* = .169, with two‐thirds to three‐quarters of caregivers at both sites being female. There were significant differences in caregivers' romantic relationship status between sites, *χ* ^2^ (3) = 19.305, *p* < .001. While most caregivers in both the United States and Mexico were married or partnered, there was a larger number of single Mexican caregivers. Education levels between sites showed significant differences, *χ* ^2^(6) = 127.113, *p* < .001, with over half of United States caregivers having post‐high school degrees and over half of the Mexican caregivers having only an elementary school education. Self‐reported social class also showed significant differences between caregivers at the Mexico and United States sites, *χ* ^2^(4) = 55.809, *p* < .001, with most United States caregivers endorsing membership in the upper middle class while Mexican caregiver membership was more evenly distributed among working class, lower middle class, and upper middle class. Caregivers' relationships to the individual with PD were significantly different between sites, *χ* ^2^ (6) = 55.919, *p* < .001. The great majority of United States caregivers were spouses or romantic partners of the individual with PD, and while over 50% of the Mexican caregivers were also spouses or romantic partners, almost one‐third were children of the individuals with PD, with other relatives comprising approximately 10% of the remainder of caregivers. The number of individuals who assisted the caregiver in providing care did not differ significantly between sites, *F*(1, 251) = 1.761, *p* = .186. There were no differences between sites in length of time (in months) providing care, *F*(1, 246) = 0.455, *p* = .501, as caregivers at both sites had provided care for an average of close to 4 years. However, caregivers at the Mexico site spent significantly more hours per week providing care than those at the United States site, *F*(1, 247) = 35.249, *p* < .001, with Mexican caregivers providing an average of an additional 48 hr of care per week compared to United States caregivers. Employment status between sites showed significant differences, *χ* ^2^ (6) = 115.766, *p* < .001; approximately two‐thirds of United States caregivers were retired, while about 40% of Mexican caregivers were employed either full‐ or part‐time, and Mexican caregivers were more likely to be unemployed or in the “other” occupational category compared to United States caregivers.

Care recipient characteristics also differed significantly between United States and Mexico sites: United States individuals with PD were older than their Mexican counterparts, *F*(1, 251) = 22.672, *p* < .001; there were more males with PD at the United States site, while the ratio between males and females was more evenly distributed at the Mexico site, *χ* ^2^ (1) = 4.072, *p* = .044; United States individuals with PD had had a diagnosis longer than the individuals at the Mexico site, *F*(1, 250) = 10.279, *p* = .002.

### 3.2 Caregiver burden differences

Results indicated that there were significant differences in personal strain between caregivers at the United States site (*M* = 10.30, *SD* = 6.85) and those at the Mexico site (*M* = 6.30, *SD* = 7.00), such that caregivers from the United States reported more personal strain, *F*(1, 251) = 20.48, *p* < .001. Similarly, there were significant differences in levels of role strain, *F*(1, 251) = 23.64, *p* < .001, between the United States site (*M* = 4.22, *SD* = 2.39) and the Mexico site (*M* = 2.68, *SD* = 2.54).

To ensure these differences in caregiver burden were not due to the significant differences in caregiver and care recipient demographic variables between sites, two ANCOVAs were conducted, controlling for all variables shown to differ significantly by site. Results indicated that the significant difference in personal strain persisted, even with these covariates, *F*(11, 236) = 14.92, *p* < .001: Caregivers at the United States site (*M* = 10.96, *SE* = 0.90) had higher levels of personal strain than caregivers at the Mexico site (*M* = 5.85, *SE* = 0.69). Similarly, the significant difference in role strain persisted with the inclusion of the covariates, *F*(11, 236) = 27.29, *p* < .001: caregivers at the United States site (*M* = 7.77, *SE* = 0.32) had higher levels of role strain than caregivers at the Mexico site (*M* = 5.31, *SE* = 0.25). For further analyses of the relationships between demographic characteristics and caregiver burden in these data, please see Smith, Perrin, Tyler, Lageman, and Villasenor (2019), which found that higher caregiver education was tied to greater burden.

## 4 DISCUSSION

The current study examined differences in caregiver burden among caregivers of individuals with PD in Richmond, Virginia, and Guadalajara, Mexico. Caregivers at the two study sites differed on a number of demographic characteristics, including age, romantic relationship status, education level, social class, caregiver relationship to the individual with PD, hours per week providing care, and employment status. Care recipients also differed demographically by age, sex, and time since diagnosis.

The finding that caregivers at the United States site were older than those at the Mexico site may be partially explained by the relationship to the individual they provided care for. For example, at the United States site, 93.3% of caregivers were spouses, while only 51.4% were spouses at the Mexico site (this might also account for the differences in romantic relationship status of caregivers). Children of the individuals with PD at the Mexico site made up 34.5% of caregivers, suggesting they would be younger in age. Age and relationship to the individual with PD differences between sites are also likely connected to the employment status differences, with about two‐thirds of the United States caregivers comprising retirees and the most frequently reported employment status in Mexico being part‐time employment (28.4%) followed by unemployment (22.3%). It is unclear if this is due to economic conditions or not being able to work full‐time (or at all) due to caregiving duties. The latter may be a plausible explanation, particularly in light of the substantial amount of time caregivers from the Mexico site reported providing care, which may preclude an individual's ability to maintain employment outside of the home. The difference in caregiver relationship to the care recipient may in part be explained by cultural values and norms. In Mexico, for example, individuals may be more likely to live in multigenerational homes, which may promote caregiving of parents.

In addition, *marianismo* and *familismo* may promote caregiving among women and other family members in Latin America. Given that *marianismo* encompasses a sense of responsibility to one's family and submissiveness to a woman's male spouse (Hubbell, 1993), it has been postulated that *marianismo* contributes to the sense of duty to care for family members (Mendez‐Luck & Anthony, 2016). This may be particularly relevant for PD caregiving, as a greater proportion of individuals with PD are men (Van Den Eeden et al., 2003), suggesting that there may be a higher proportion of female caregivers in Latin America compared to other geographic regions due to the prevalence of spousal caregiving.

Similarly, the concept of *familismo* (familism) may influence roles, obligations, and expectations within families in Latin America (Zea, Quezada, & Belgrave, 1994). *Familismo* also emphasizes the importance of caring for one's family and their needs over one's own needs, as well as a respect for older individuals in the family (Ruiz & Ransford, 2012). Latino informal caregivers are less likely to institutionalize the individual they provide care for (Dilworth‐Anderson, Williams, & Gibson, 2002) and are less likely to use formal support services (Dilworth‐Anderson et al., 2002; Pinquart & Sörensen, 2005). *Familismo* may also be associated with positive outcomes in the caregiving context. For example, higher levels of *familismo* were associated with lower burden among one sample of Latino caregivers (Coon et al., 2004). When compared to individuals of other racial/ethnic groups, Latino informal caregivers have less desire to stop providing care and are more satisfied in their role as a caregiver (Phillips, de Ardon, Komnenich, Killeen, & Rusinak, 2000). Further, *familismo* may be a protective factor as other family members may be more likely to support the primary caregiver.

Caregivers from the Mexico site reported spending significantly more hours providing care each week than those from the United States site. This tendency seems to hold true in general, as a review of racial/ethnic differences after traumatic brain injury also found that Hispanic caregivers spent more time caregiving than White caregivers (Gary, Arango‐Lasprilla, & Stevens, 2009). Time spent caring in the current sample of caregivers was higher than other samples of caregivers of individuals with neurological conditions in Mexico. For example, a sample of caregivers of individuals with multiple sclerosis in Guadalajara, Mexico reported on average spending 70.96 hr a week providing care (Mickens et al., 2018), nearly 40 hr less than caregivers from the current sample. A more thorough investigation of caregiving activities among PD caregivers in Mexico may serve to explain the number of hours spent caregiving. The majority of studies on PD caregivers have found no (Martinez‐Martin et al., 2008; Martínez‐Martín et al., 2007; Shin, Lee, Youn, Kim, & Cho, 2012) or only weak associations (Kim et al., 2007; Razali, Ahmad, Rahman, Midin, & Sidi, 2011; Tew, Naismith, Pereira, & Lewis, 2013) between the amount of hours spent providing care and caregiver burden.

There are several potential reasons why caregivers in the United States sample reported greater levels of burden, despite caregivers in Mexico being at significant disadvantage across a number of demographic indices. At least one study has found that burden is greater among spousal caregivers (Viwattanakulvanid et al., 2014), which may partially explain the differences in caregiver burden, as over 90% of caregivers at the United States site were spouses while only 51.4% of caregivers at the Mexico site were spouses. However, the differences in burden in the current study still remained even when controlling for caregiver relationship to patient. It is also possible that caregivers in Mexico experienced lower burden as a result of providing care in the context of cultural factors such as *marianismo*, *familismo*, and *respeto* (respect). These cultural values encourage the importance of caring for the family as well as respecting elders (Neary & Mahoney, 2005), which may promote caregiving and even make it a point of cultural pride. As such, individuals at the Mexico site may view the opportunity to care for their loved one as meaningful and gratifying instead of burdensome.

### 4.1 Limitations and future studies

This study was conducted in only two urban PD outpatient clinics. As such, it may not be generalizable to PD caregivers in rural or suburban areas. As individuals in the later stages of PD are more likely to be institutionalized and caregiver burden is likely to be highest immediately preinstitutionalization (Deloitte Access Economics, 2015), this study may not have captured the experiences of caregivers of individuals in the later stages of PD. Likewise, future studies should differentiate among caregivers of those individuals with PD who have developed dementia and those who have not, as cognitive and behavioral symptoms exhibited by individuals with dementia in Colombia have been linked to higher levels of caregiver burden (Arango Lasprilla, Moreno, Rogers, & Francis, 2009).

Data were collected by asking United States caregivers to complete written questionnaires independently, but at the Mexico site, researchers used oral interviews to collect information from participants. Therefore, it is possible that this may have influenced the way participants answered questions. Future studies should investigate the nature of caregiving tasks performed by PD caregivers, the time spent on each task, and the caregiver's perception of burden related to those tasks to further delineate possible cultural differences in appraisal of burden. An investigation of caregiver burden in low‐income female caregivers in Mexico found both positive and negative attributions for caregiver burden in many participants (Mendez‐Luck, Kennedy, & Wallace, 2008).

Finally, while the current study examined cross‐cultural differences in caregivers' demographic characteristics and a previous article (Smith et al., 2019) examined the contributions of these demographics to burden, future studies would benefit from examining the relationships between the demographics and burden across the two countries in advanced statistical models (e.g., invariance models of caregiving characteristics and burden). Cultural values and/or norms might have contributed to the site differences in caregiver demographics and burden found in the current study, and variables reflecting the role of culture were not included; by extension, cultural variables were not examined in invariance models that might differentially explain relations between demographics in burden between the United States and Mexico. If cross‐national differences underscore the importance of culture, future studies could include additional statistical analyses with cultural value variables as covariates of or explanations for possible differences.

## 5 CONCLUSION

The results of the current study found that though there were significant differences in demographic variables for both caregivers and care recipients between the United States and Mexico sites, these demographic variables did not account for the differences in reported PD caregiver burden. Despite considerably more time spent in caregiving duties, higher rates in unemployment or underemployment, and lower education levels, Mexican PD caregivers reported significantly less personal strain and role strain than did their United States counterparts. The scientific and medical communities should view caregiving as a culturally embedded and potentially positive role, rather than predominantly as burdensome as frequently conceptualized in Western or Eurocentric cultures.

## CONFLICTS OF INTERESTS

The authors have no conflicts of interests to declare.

## AUTHOR CONTRIBUTION

ERS, PBP, SKL, and TV involved in study design. ERS, SKL, and TV collected the data. ERS and PBP drafted the manuscript. ERS, PBP, and CMT statistically analyzed. PBP, CMT, SKL, and TV edited the manuscript.

### Peer Review

The peer review history for this article is available at https://publons.com/publon/10.1002/brb3.1753.

## Data Availability

Data from this study are available by request from the corresponding author.
